# Open access: academic publishing and its implications for knowledge equity in Kenya

**DOI:** 10.1186/1744-8603-10-26

**Published:** 2014-04-09

**Authors:** Duncan Mwangangi Matheka, Joseph Nderitu, Daniel Mutonga, Mary Iwaret Otiti, Karen Siegel, Alessandro Rhyll Demaio

**Affiliations:** 1 Department of Medical Physiology, School of Medicine, University of Nairobi, PO Box 30197–00100, Nairobi, Kenya; 2 Young Professionals Chronic Disease Network, Boston, USA; 3 Department of Human Anatomy, School of Medicine, University of Nairobi, Nairobi, Kenya; 4 Nutrition and Health Sciences, Graduate Division of Biological and Biomedical Sciences, Laney Graduate School and Department of Global Health, Rollins School of Public Health, Emory University, Atlanta, GA, USA; 5 Department of International Health, Immunology and Microbiology, Copenhagen School of Global Health, University of Copenhagen, Copenhagen, Denmark; 6 Harvard Global Equity Initiative, Department of Global Health and Social Medicine, Harvard Medical School, Boston, USA

**Keywords:** Traditional publishing, Open access, Kenya, Low- and middle-income countries, Repositories, HINARI, Policy, Libraries, Universities, Open access week

## Abstract

Traditional, subscription-based scientific publishing has its limitations: often, articles are inaccessible to the majority of researchers in low- and middle-income countries (LMICs), where journal subscriptions or one-time access fees are cost-prohibitive. Open access (OA) publishing, in which journals provide online access to articles free of charge, breaks this barrier and allows unrestricted access to scientific and scholarly information to researchers all over the globe. At the same time, one major limitation to OA is a high publishing cost that is placed on authors. Following recent developments to OA publishing policies in the UK and even LMICs, this article highlights the current status and future challenges of OA in Africa. We place particular emphasis on Kenya, where multidisciplinary efforts to improve access have been established. We note that these efforts in Kenya can be further strengthened and potentially replicated in other African countries, with the goal of elevating the visibility of African research and improving access for African researchers to global research, and, ultimately, bring social and economic benefits to the region. We (1) offer recommendations for overcoming the challenges of implementing OA in Africa and (2) call for urgent action by African governments to follow the suit of high-income countries like the UK and Australia, mandating OA for publicly-funded research in their region and supporting future research into how OA might bring social and economic benefits to Africa.

## Introduction

Traditionally, scientific discoveries are shared primarily through subscription-based journals and ‘fee-to-access’ publications. This makes access to such scholarly literature inequitable, since access to science is based on the user’s ability to pay [1]. Research findings locked behind these “pay-walls” are not disseminated as widely as possible, leading to restricted readership and thus diminishing their overall impact [2-4]. Open Access (OA) publishing attempts to address this dilemma [2,5,6].

OA is the free, immediate, online availability of peer-reviewed research articles with full re-use rights [7,8]. OA is more equitable than traditional scientific publishing and allows anyone with an internet connection, regardless of where they are in the world, to access, read, and build upon the most up-to-date scientific literature [7,8]. It has the potential to accelerate additional discoveries, knowledge sharing and improve education [9]. Despite a number of challenges (including a frequently high publishing cost that is placed on the authors, not the readers), many scientists and researchers around the world see the value of OA and are working to increase its use and to reform the traditional publishing processes (the status-quo) [10,11].

OA forms also known as the roads or routes to OA include: (1) OA journals (the “Gold” road to OA), and (2) self-archiving in OA repositories (the “Green” road to OA) [5,12,13]. OA journals (the Gold road) do not charge users. They are peer-reviewed and published research papers, free of any access charge and with maximum opportunities for re-use. Instead, they look to other sources to fund peer-review and publication costs, including charging article processing charges (APCs) to the authors [5,14]. The green route includes self-archiving in OA repositories. In self-archiving, researchers simply deposit their refereed journal articles in open electronic archives which conform to Open Archives Initiative standards. OA repositories (Green) are online databases providing free access to the full text of research papers and other types of material. OA repositories generally host articles peer-reviewed elsewhere. OA repositories can include preprints and postprints of journal articles, theses and dissertations, course materials, departmental databases, data files, audio and video files, institutional records, or digitized special collections from the library [5,8,12,13]. Self-archiving sustains OA repositories and this strategy helps researchers to avail their work to others faster by bypassing the rigors of journal publishing.

OA is becoming increasingly encouraged, even mandated, in some high-income countries (HICs). For example, the European Union (EU) runs a project to encourage all EU-funded research to place results in an OA repository and publish in OA journals [15]. At the same time, the Wellcome Trust and some countries, including the United Kingdom’s (UK’s) Research Council [14,16], Denmark and Australia [17], are either planning or implementing OA policies whereby the publication of publicly funded research in OA journals will be mandatory. These have been beneficial, with OA articles being cited earlier and more often than non-OA articles [6,13-17]. This demonstrates potential for OA to accelerate and improve scientific advancement, research visibility, knowledge dissemination, and translation of knowledge from research into practice.

These developments in HICs have the potential to impact African researchers both positively and negatively. On the one hand, scientific researchers in the African region may soon be able to access more research articles than they were previously able to. On the other hand, these same individuals may not be able to publish in gold OA journals due to the high publishing cost and the fact that most African governments, including Kenya, have no explicit policies mandating OA publishing akin to the one in UK [1,14-17].

### Overview of access to research publications in Africa

There are currently two key challenges to equitable access to scientific research publications in Africa. The first is the lack of access to subscription-based scientific journals. Traditionally, when scientific breakthroughs relevant to Africa are made, they are published in non-OA journals whose prohibitive subscription (or access) costs are often in the order of US$30 per paper (The price per article for Elsevier journals on ScienceDirect is US$31.50 as of November 11, 2013) [18], which is unaffordable for most researchers in LMICs [19].

The high subscription cost inhibits the exposure of African research scientists to these discoveries and their ability to either use the most up-to-date research knowledge to strengthen their research or to build upon said research [19-21]. This can lead to additional adverse consequences on education and research in Africa, which leads to a perpetuated cycle of restricted knowledge. African research scientists might lack access to their fellow researchers’ work, and students may lack access to the work of their senior colleagues and researchers around the world. Researchers and students therefore lack capacity to practice evidence-based science confounding further to the challenges in implementing research projects and translating research findings into marketable products [13,22].

To curb the costs of journal subscriptions, the World Health Organization (WHO), through Health Inter Network Access to Research Initiative (HINARI), facilitates free access to over 3,000 journals in most LMICs [23]. Through HINARI, publishers give institutions in LMICs free access to electronic editions of journals. This is through a WHO-funded platform that can be accessed via a username and a password – which are available among researchers in LMICs [23-25]. Moreover, programs such as authorAID workshops exist, which encourage research and facilitate the process for authors [26].

OA journals also address these access issues, and a number of OA research journals have been launched in Africa, with South Africa being on the lead [1,27-31]. Scholarly Communication in Africa Program (SCAP) also aims to increase the visibility of African research via research journals as well as other ways of disseminating such work [31].

However, these OA journals raise the second key challenge to equitable access to research and knowledge sharing in Africa: to offset the fact that OA journals have no subscription costs, they shift this burden to authors, charging high publishing fees ranging from US$1000 to US$2500 per publication [1,21]. This precludes African research scientists from being able to publish their findings in these OA journals. To address this, some OA journals currently offer APC waivers to researchers who cannot cover OA journals’ publishing costs. Public Library of Science (PLOS) [32] and BioMed Central (BMC) [10] are both examples of publishers with automatic waivers for researchers based in LMICs, as classified by the World Bank [1]. Researchers in a country on the waiver list would not face any financial barrier when publishing in these OA journals [1].

### Overview of OA initiatives in Kenya

Although OA is still burgeoning in Africa, several established initiatives exist, such as the following, in Kenya.

#### Role of libraries

Through the Kenya Library and Information Services Consortium (KLISC), libraries in Kenya are at the forefront advocating for OA journal publishing and policies [33,34]. KLISC was formed in 2004, when Kenyan public universities came together with the help of International Network for the Availability of Scientific Publications (INASP) [35]. Its current membership includes Kenyan private universities, public libraries, museums and research institutions with the aim of ensuring access to electronic resources, including OA materials [34].

Kenyan libraries are also members of the Programme for Enhancement of Research Information (PERii) since 2001. PERii promotes OA through compiling a list of OA resources, promoting OA repositories and supporting publishing – for example, Africa Journals Online (AJOL) and authorAID Project [26,30]. The Electronic Information for Libraries (EIFL), has also catapulted the drive for OA in Kenya [33,34,36].

The librarians of the University of Nairobi have significantly contributed in ensuring the adoption of an OA policy by the University. This was accomplished through a series of trainings to the dons of the university on the role of OA in advancement of research, and associated benefits for the institution [37].

#### Open access research journals

There are a number of OA research journals that have been launched in Kenya, mainly run by Kenyan institutions such as Kenya Medical Research Institute, University of Nairobi, among others [28,29]. These are often free to the researcher as well as the reader, with the publishing costs being incurred by the hosting institutions. However, there are challenges faced in the quality of such journals, as well as their peer review process, relative impact factors and sustainability.

#### Universities and other institutions

Various institutions in Kenya have adopted OA policies making research work carried out by these institutions openly accessible. They run e-repositories where research conducted by their students and faculty is deposited [37]. Some universities and institutions are already running OA journals too [28,29].

OA policies have already been adopted by several Kenyan institutions including Strathmore University, Multimedia University, University of Nairobi, Kenyatta University, Jomo Kenyatta University of Agriculture and Technology, African Population and Health Research Centre, among others [33,34].

Moreover, most Kenyan institutions now ensure access to wireless internet connection throughout their facilities. This is an important step in creating a framework to take full advantage of OA and to create and sustain OA repositories.

#### Students and open access week

Every year, there is a global OA Week [38] – an event to bring attention to this rapidly emerging form of scientific publication. In Kenya, the University of Nairobi and Jomo Kenyatta University of Agriculture and Technology libraries and students have over the years used this week to create more OA awareness in their institutions [35,39].

Through the Medical Students’ Association of Kenya (MSAKE), a member of the International Federation of Medical Students’ Association, this week has been marked by students in all public medical schools in Kenya (University of Nairobi, Moi University, Kenyatta University and Egerton University) in 2012 [1,35]. MSAKE also runs a blog and social media websites all year round as a tool for advocacy and creating OA awareness [40].

#### Kenyan government

Like most African governments, the Kenyan government has no explicit policies mandating OA publishing akin to those in the UK and Australia [14-17]. However, it has made steps in funding local research through its National Commission for Science, Technology and Innovation. Through this commission, grants have been administered to support scientific research and innovations for national development, targeting priority areas in relation to the development agenda of the government. Since inception of this program in 2008/2009, it has stimulated renewed interest among researchers and innovators in various institutions across the country [41].

### Recommendations for open access in Africa

Besides the advances made on OA in Kenya, a number of constraints to equitable access to scientific research publications still exist. Like other LMICs, Kenya lacks access to expensive subscription-based scientific journals and OA policies, and researchers are burdened by high publishing fees. Therefore, it is prudent to strengthen the efforts in Kenya, and potentially replicate in other African nations.

OA implementation requires multidisciplinary input and therefore needs various stakeholders (governments, researchers, institutions, journals, libraries, civil society, non-governmental organizations (NGOs), policymakers, Information and communications technology (ICT), students) to work collaboratively to solve these challenges.

#### Government

An important step for African governments would be to implement policies to ensure that research findings are shared widely, through public funding for OA publishing costs. Additionally, there should be legislative measures mandating OA publishing for all research pioneered by African governments or that which is publicly funded. The EU launched its bid to have all EU-funded work to be made available to researchers without discrimination [14-17]. It is necessary for African governments to follow this example since this will be an important catalyst to scientific innovation in the region. Government funding for medical research is either lacking or minimal in African countries. A big step would be for the African governments to establish or increase funding for medical research which would help cover the publishing cost.

#### Researchers

African researchers should support OA by publishing their research in OA journals, and/or deposit their peer-reviewed manuscripts in OA repositories. This will ensure equity and a benefit to the people who funded the work, as well as to fellow researchers.

#### Non-government sector

Institutions, libraries, civil society and NGO policy makers should also be at the forefront in embracing OA. They need to encourage more research and publishing. Moreover, OA policies should be adopted by institutions in African countries, as done by institutions in HICs - Harvard University, Stanford University, National Institutes of Health, Wellcome Trust, Canadian Cancer Society, Autism Speaks, and Canadian Institutes of Health Research [16,42-44].

#### Advocacy

Advocacy for OA is crucial towards securing the support by universities, governments, research funders, charitable funders and other organizations. This should also be aimed at more journals giving waivers to LMICs or those who cannot afford APCs. Like in Kenya, students also have a role to play in advocacy for OA and even for necessary infrastructure and policies.

#### Journals

There is a role to encourage establishment of more OA journals in Africa, as well as committing more resources to ensure better quality, peer review and sustainability of these journals. For the traditional journals, there is a role for their retrospective digitalization and integration with existing ICT. Through this, the ‘traditional’ academic journals can be incentivized to change their business models and make scientific knowledge open to all.

#### Research funding bodies

Lastly, there is need to find a solution to the dilemma of funding for publications from LMICs. Can LMIC governments fund publishing costs? Can researchers and LMICs liaise with more publishers to waiver APCs for researchers from LMICs? Can there be constant funders to support publication fees for researchers from LMIC (just like the WHO-pioneered HINARI for access to journals)? or Can publishing fees be abolished?

## Conclusions

Open Access is crucial for the process of informing the scientific community on research outcomes since it ensures quicker, more direct access to research results and reduces inequities in scientific access. With Africa currently facing serious challenges in research and publishing, multidisciplinary support mandating OA publication is urgently needed - a move that is likely to bring social and economic benefits in Africa and elevate the visibility of African research around the world. We note that the multidisciplinary efforts in Kenya can be strengthened and potentially replicated in other African countries. We recommend that the African governments mandate OA for publicly funded research in their region akin to UK and Australia, as well as support research into the economic and social benefits of OA for the African region.

## Abbreviations

APCs: Article processing charges; EU: European union; HINARI: Health inter network access to research initiative; HICs: High-income countries; KLISC: Kenya library and information services consortium; LMICs: Low- and middle-income countries; NGOs: Non-governmental organizations; OA: Open access; UK: United Kingdom; WHO: World Health Organization.

## Competing interests

The authors declare that they have no competing interests.

## Author’s contributions

DMM conceived and wrote the first draft of the manuscript. DM MO KS and AD contributed significantly to the writing and review of the manuscript. All authors read and approved the final manuscript.
